# Clinical validation of robot simulation of toothbrushing - comparative plaque removal efficacy

**DOI:** 10.1186/1472-6831-14-82

**Published:** 2014-07-04

**Authors:** Tomas Lang, Sebastian Staufer, Barbara Jennes, Peter Gaengler

**Affiliations:** 1 ORMED Institute for Oral Medicine, University of Witten/Herdecke, Alfred-Herrhausen-Str. 45, 58455 Witten, Germany; 2 Faculty of Health, Department of Dentistry, University of Witten/Herdecke, Alfred-Herrhausen-Str. 50, 58448 Witten, Germany

**Keywords:** Toothbrushing, Plaque control, Cleaning efficacy, Robot simulation, Randomized clinical trial, Oral hygiene

## Abstract

**Background:**

Clinical validation of laboratory toothbrushing tests has important advantages. It was, therefore, the aim to demonstrate correlation of tooth cleaning efficiency of a new robot brushing simulation technique with clinical plaque removal.

**Methods:**

Clinical programme: 27 subjects received dental cleaning prior to 3-day-plaque-regrowth-interval. Plaque was stained, photographically documented and scored using planimetrical index. Subjects brushed teeth 33–47 with three techniques (horizontal, rotating, vertical), each for 20s buccally and for 20s orally in 3 consecutive intervals. The force was calibrated, the brushing technique was video supported. Two different brushes were randomly assigned to the subject. Robot programme: Clinical brushing programmes were transfered to a 6-axis-robot. Artificial teeth 33–47 were covered with plaque-simulating substrate. All brushing techniques were repeated 7 times, results were scored according to clinical planimetry. All data underwent statistical analysis by t-test, U-test and multivariate analysis.

**Results:**

The individual clinical cleaning patterns are well reproduced by the robot programmes. Differences in plaque removal are statistically significant for the two brushes, reproduced in clinical and robot data. Multivariate analysis confirms the higher cleaning efficiency for anterior teeth and for the buccal sites.

**Conclusions:**

The robot tooth brushing simulation programme showed good correlation with clinically standardized tooth brushing.

This new robot brushing simulation programme can be used for rapid, reproducible laboratory testing of tooth cleaning.

## Background

Plaque removal by manual or powered toothbrushing is still the most effective preventive method to control gingivitis manifestation and caries lesion stagnation or progression [1]. This cornerstone of oral hygiene is supported by lifelong local applications of different fluoride formulations and, when needed, of various antibacterial agents.

To motivate consumer’s oral hygiene behaviour and to enhance the patient’s compliance towards the recommended tooth cleaning efficacy, new toothbrush designs are permanently developed and tested. In contrast to the past, no “standard” toothbrush is dominating the market today. Preferences concerning handle configuration and brushhead design differ widely among preventive caregivers and consumers. Different age groups, patient’s disease profiles, patients in special needs etc. require individual toothbrush models and, consequently, individual brushing techniques in the frame of contemporary personalized preventive medicine. New toothbrush models for this various target groups should remove plaque equally efficient or better than their predecessors and, therefore, their plaque removal efficacy needs to be tested prior to manufacture.

The ultimate goal of such testing would be the outcome of clinical testing under field conditions by assessing full mouth plaque removal and gingivitis scores. This is, however, very time consuming, rather expensive and difficult to standardize for later comparative meta-analyses [1]. Therefore, the assessment of in vitro tooth cleaning efficacy became a real alternative to clinical trials in testing many different designs and action modalities of manual and powered toothbrushes. Since 1972 several test environments were developed to test manual and powered toothbrushes prior to manufacture or clinical testing. Arnold and Trost were the first to introduce a simple brushing machine using horizontal movements on acrylic tooth models covered with a water-based dye [2]. A more sophisticated equipment by Nygaard-Østby et al. was primarily developed to measure inter-proximal penetration of the toothbrush bristles by using a typewriter colour ribbon band to simulate inter-proximal space during horizontal or vertical brushing movements with brushing forces between 2.5 and 10.0 N [3]. Rawls et al. proposed static and dynamic tests using recommended brushing techniques on blue ethyl cellulose coated typodont models at an angulation of 45°. Moistened toothbrushes were applied with weight controlled force between 1.0 and 10.0 N [4]. The interproximal penetration of bristles was controlled and measured by a high-speed video camera and a colour removal index. The disadvantage of the experimental approach was the colour coating of plastic teeth not simulating the adherence of plaque biofilms on natural teeth. Therefore, Volpenhein et al. developed a plaque simulating red coating based on ethyl ester and copolymer. Manual toothbrushes were moved in two angulations (45° and 90º) and three directions (horizontal, vertical and rotating) over typodont models [5]. Cleaning efficacy was assessed by the coating removal at a 10fold magnification of plastic teeth. According to the variability of the experimental protocol, this device was the first robot-like in vitro approach.

The first 6-axis robot was used to simulate the 3-dimensional brushing patterns of powered toothbrushes. The typodont models were stained with a water-based colour and brushed for 1 minute. A modified plaque index was used by two blinded examiners to score the results [6,7]. The tooth cleaning efficacy assessment was later improved by a computerized vision system [8]. Recently, the robot test of brush head wear was used to assess the area covered with plaque substitute by a 3 D laser system [9]. Since a decade the brushing machine of Imfeld et al. is a well-established method of assessing in vitro tooth cleaning efficacy of manual and electric toothbrushes [10-12]. Test brushes were mounted on an automated brushing machine, which moved over a custom-made tooth model of a posterior or anterior segment. All black tooth surfaces were coated with white titanium oxide in ethanol to simulate complete plaque accumulation. Tooth surfaces reappearing black after brushing were regarded as cleaned, digitized and planimetrically analyzed.

The Navy-Plaque-Index was introduced by Elliot et al. to support the assessment of plaque in clinical studies by sectioning the buccal tooth surface into six areas (three gingival, and each one mesial, distal and incisal area) [13]. Based on the need to test oral hygiene products the Navy-Plaque-Index was modified by Rustogi et al. by adding three additional zones above the gumline and below the equatorial line [14]. Claydon and Addy modified this refined index by using planimetry for the examination process [15]. After plaque revelation standardized clinical photographs were used to score all teeth buccally and orally with 576 planimetrical fields per subject. This resulted in better differentiation of plaque removal per tooth. Therefore, this planimetrical index is now well established, because of the reproducibility, blinded assessment and safe documentation.

In summary, there are sophisticated in vitro tooth cleaning methodologies, however, none of them have been clinically validated. And there are well established clinical index systems assessing reproducible plaque scores. It was, therefore, the aim of the present study to bridge the gap between the clinical performance and plaque removing outcome of tooth brushing and the in vitro simulation by the extraordinary programme flexibility of a six-axis robot.

## Methods

### Clinical programme

**Figure 1:**
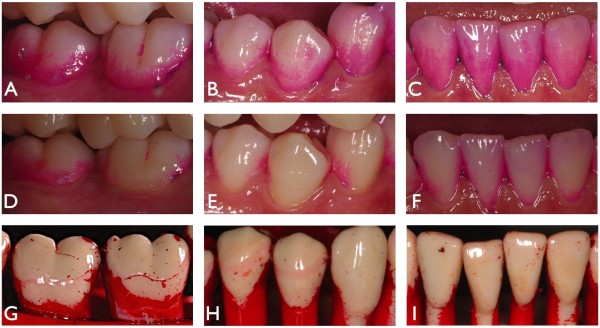
**Example of photographic documentation of the clinical programme (A-F) and the robot programme (G-I). A**-**C**: stained plaque after 3-day plaque regrowth. **D**-**F**: same teeth, stained plaque after 20s of toothbrushing. **G**-**I**: typodont with simulated plaque after toothbrushing.

**Figure 2:**
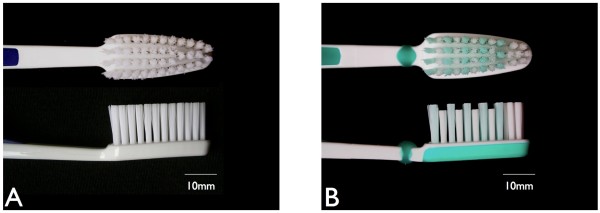
**Tested toothbrushes. A**: Dr.Best® plus medium. **B**: Dr.Best® Interdent medium (GlaxoSmithKline, Buehl, Germany).

Approval for the clinical study was provided by the Ethics committee of the University of Witten/Herdecke (Application No. 55/2007) and was conducted according to the guidelines for Good Clinical Practice. Subjects were given verbal and written information concerning the aim of the study and they gave signed consent to participate. The study was a randomized, 3 periods, single blind, parallel design trial involving 27 highly trained undergraduate dental students (12 male, 15 female, age 19–28 years). The primary aim was the planimetrical plaque removal efficacy of 2 test toothbrushes in 3 consecutive brushing movements (horizontally, rotating, vertically) at a brushing force of 3.5 N in this clinical programme and the transfer of all relevant brushing conditions to the robot programme. The volunteers were recruited according to the inclusion criteria: The subjects were medically fit, did not have orthodontic appliances or removable dental prostheses, and had sound caries-free and periodontitis-free teeth (lower incisors and right lower premolars and molars).All subjects received before each of the 3 study periods a professional dental cleaning prior to a 3-day plaque-regrowth-interval. Plaque was stained and photographically documented before (Figure 1A-C) and after tooth brushing (Figure 1D-F). A videoclip was presented to each subject showing the recommended brushing technique prior to testing. The brushing force of 3.5 N was calibrated by brushing a typodont model mounted on a weight prior to each test. While brushing their teeth the brushing technique was video supported by showing the same mirrored videoclip used for calibration. The subjects brushed their teeth 33–47 with the three most recommended brushing techniques (horizontally, rotating, vertically), each for 20 s buccally and for 20 s orally, and in 3 consecutive study periods. After a wash-out interval of 4 days all subjects continued with the next brushing technique starting with a professional cleaning and a 3-day plaque-regrowth-interval.Two different toothbrushes were compared. The Dr.Best® plus medium, which has a flat trim and consists of 43 individual tufts (number of subjects = 13) and the Dr.Best® Interdent medium (GlaxoSmithKline, Bühl, Germany) which has an interdental cut and consists of 42 individual tufts (number of subjects = 14). Both brush heads had nearly the same size (Figure 2).

### Robot programme

**Figure 3:**
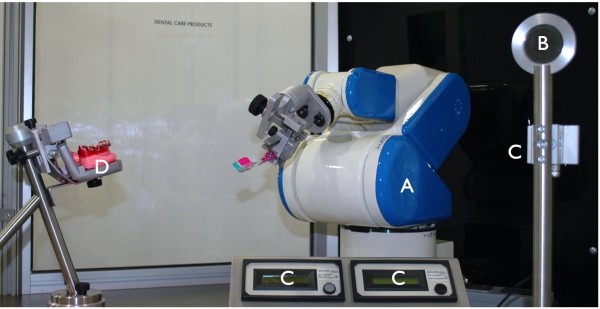
**Toothbrushing simulation unit. A**: six-axis robot, **B**: calibrating graticule. **C**: two shields for calibrating the brushing force. **D**: mounting plate for mandibular typodont dentition 33–48.

The primary aim of the robot programme was the meticulous robot teaching according to all standardized clinical parameters to compare the plaque removal efficacy as evidence of clinical validation. The clinical brushing programmes were meticulously transferred to the 6-axis-robot FS 02 N (Kawasaki Robotics, Akashi, Hyogo, Japan) so that the brushing force and time, the angulation and movements reflect the parameters of the clinical trial (Figure 3). The artificial teeth 33–47 (KaVo, Biberach, Germany) were covered with a plaque simulating substrate. To ensure equal consistency and thickness of the simulated plaque-film a special second robot was developed to cover the teeth and dry the substrate automatically. For every cycle a new set of typodont teeth (D) and a new toothbrush were used. The toothbrush was inserted in an individual mount to ensure a proper and tight fit. Then the toothbrush was centered in all spatial axes on the calibrating graticule (B). Finally the brushing force was calibrated to a total of 3.5 N on two separated shields (C) to ensure the same amount of brushing force at the distal and proximal side of the brush-head (Figure 3A-D). All three brushing techniques were repeated seven times for both toothbrush models, and cleaning results were photographically documented with the same equipment used in the clinical programme. The photo documentation repeated the same clinical procedures, the angulation of the camera and the mirror placement (Figure 1G-I). Constant room temperature and humidity were established during the laboratory tests. The exact robot operation is constantly supervised by Kawasaki service team. The complete testing process and the SOP’s were independently evaluated and approved by the German State Material Testing Agency [16].

### Scoring

The photographs of the clinical and robot programmes were categorized, blinded and scored by a calibrated examiner using the planimetrical index [15], with 9 fields at buccal sites and 9 fields at oral sites of all teeth (score 0 - no plaque; score 1 - plaque or plaque residues present).

### Statistics

The results of the planimetric scoring underwent statistical analysis by t-test, F-test and Wilcoxon signed-rank test. The outliers were detected prior to the analysis with the Grubbs’ test for outliers. To deal with the inhomogeneity of the sample values (n^1^ = 27, n^2^ = 14) a modification of the t-test (SATTERTHWAITE-WELCH-Test) was used to calculate the mean values of residual plaque between the subjects and the robot. The correlation of plaque removal efficacy of clinical and robot testing was detected with the Spearman rank correlation coefficient, and cleaning differences between the 3 brushing movements and the 2 toothbrushes were tested by multivariate analyses (see [17] for nonparametric statistics and [18] for t-tests and multivariate analysis).

Additional statistical tests were calculated excluding the planimetrical risk fields A, B and C next to the gumline to demonstrate the effect of gingival masks around typodonts. However, the number of significant differences in mean values of residual plaque between subjects and robot did not change, therefore, all 9 planimetrical fields per tooth site were included to calculate summary statistics of the plaque measurements for validation purposes only.

## Results

### Clinical outcome

Both tested toothbrushes removed stained plaque in all 3 brushing movements and at all examined teeth with minor statistical difference (brushing technique horizontally: lingual t = 0.03 (p > 0.10), buccal t = −1.79 (0.05 < p < 0.10); brushing technique rotating: lingual t = 0.24 (p > 0.10), buccal t = −0.89 (p > 0.10); brushing technique vertically: lingual t = −0.15 (p > 0.10), buccal t = −0.69 (p > 0.10). The mean values of the number of fields with residual plaque ranged from 1.96 to 3.81 with brushing technique horizontally, 1.59 to 4.37 with rotating and 2.00 to 4.33 with vertical brushing movement (of a total of 9.00 fields per site, all covered with plaque). The cleaning efficacy was rather tooth-specific and less site-specific. Incisors and canines were best cleaned, followed by premolars, and molars clearly exhibited the least plaque removal. Oral sites of molars were better cleaned compared to buccal sites. However, all buccal sites of premolars, canines and incisors exhibited better cleaning performance vs. oral sites (Table 1).

**Table 1:** Comparison of mean values of residual plaque (not completely cleaned planimetrical fields, the maximum score of the planimetrical plaque Index is 9 (score 1 in each field A-I, the minimal score is =0 (no residual plaque in all 9 fields at the oral or buccal site)) in subjects and in robot testing

		**Horizontal**				**Rotating**				**Vertical**			
**Tooth**		**Subject**	**Robot**	**t-value**		**Subject**	**Robot**	**t-value**		**Subject**	**Robot**	**t-value**	
47	oral	3,48	5,14	−3,78	(***)	4,11	4,50	−0,72		4,33	6,57	−5,67	(***)
	buccal	3,81	1,64	5,93	(***)	4,37	1,64	6,80	(***)	4,15	3,79	0,84	
46	oral	3,41	4,57	−2,37	(**)	3,74	3,93	−0,37		4,26	6,64	−8,67	(***)
	buccal	3,52	1,93	3,91	(***)	3,30	2,36	1,80	(*)	3,59	2,79	1,94	(*)
45	oral	3,30	1,57	3,82	(***)	3,37	1,43	4,03	(***)	3,63	6,14	−5,45	(***)
	buccal	3,41	2,21	3,32	(***)	2,89	2,00	2,09	(**)	3,15	3,50	−0,79	
44	oral	2,89	2,43	1,22		2,96	2,14	1,70	(*)	3,48	6,14	−5,16	(***)
	buccal	2,74	1,36	3,70	(***)	2,70	1,21	3,27	(***)	3,04	3,29	−0,49	
43	oral	3,00	1,71	3,78	(***)	2,78	1,29	2,81	(***)	3,07	3,43	−0,64	
	buccal	2,00	1,64	0,89		1,93	1,93	−0,01		2,11	2,79	−1,33	
42	oral	3,07	1,14	4,29	(***)	2,93	1,43	3,53	(***)	3,04	1,50	3,17	(***)
	buccal	2,15	0,57	4,56	(***)	1,85	0,57	3,85	(***)	2,00	3,29	−2,44	(**)
41	oral	2,96	1,93	2,80	(**)	2,89	2,64	0,59		2,89	2,79	0,17	
	buccal	2,37	0,21	6,49	(***)	1,81	0,64	3,40	(***)	2,44	3,50	−2,02	
31	oral	2,33	1,93	1,24		2,52	1,21	3,28	(***)	2,67	3,14	−0,72	
	buccal	2,22	0,86	3,66	(***)	1,78	1,07	1,89	(*)	2,37	2,64	−0,51	
32	oral	2,37	1,57	1,95	(*)	2,37	1,21	2,97	(***)	2,56	2,29	0,39	
	buccal	1,96	0,93	3,04	(***)	1,59	1,21	1,11		2,11	2,57	−1,06	
total	oral	26,81	22,00	2,15	(**)	27,67	19,79	2,75	(***)	29,93	38,64	−2,55	(**)
	buccal	24,19	11,36	5,17	(***)	22,22	12,64	3,48	(***)	24,96	28,14	−1,00	

Number of observations: clinical study: n = 27, robot study: n = 14.

The null hypothesis of the equity between the mean values of subject versus robot was rejected with a significance level of (*): p < 0.10, (**): p < 0.05, (***): p < 0.01.

### Robot outcome

Both tested toothbrushes removed simulated plaque coatings in all 3 brushing movements with measurable statistical differences (brushing technique horizontally: lingual t = 4.55 (p < 0.01), buccal t = 4.04 (p < 0.01); brushing technique rotating: lingual t = 2.99 (0.01 < p < 0.05), buccal t = 5.90 (p < 0.01); brushing technique vertically: lingual t = −5.34 (p < 0.01), buccal t = −5.89 (p < 0.01). The average number of fields with remaining plaque ranged from 0.21 to 5.14 (horizontally), 0.64 to 4.50 (rotating) and from 2.00 to 4.33 (vertically). All robot data are represented in Table 1. The cleaning efficacy was again mainly tooth-specific and less site-specific except the two molars. Incisors, canines and premolars were much better cleaned compared to molars. Most buccal sites, except the second premolar, exhibited less simulated plaque compared to the lingual sites.

### Robot vs. clinical plaque removal

**Figure 4:**
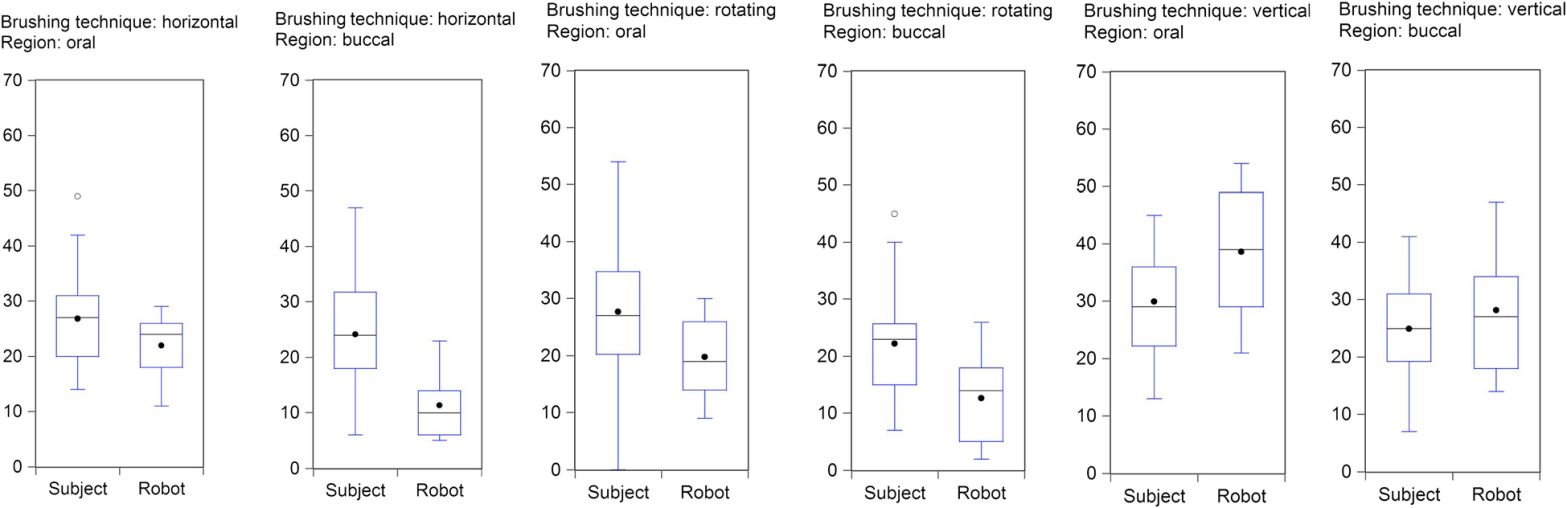
**Comparison of different brushing techniques between the robot and the subjects.** Cumulative number of not completely cleaned planimetrical fields of teeth 32–47, both toothbrushes and all subjects and robot cycles (total number of fields 9x9 = 81). Explanation: Number of observations: n = 27 subjects; n = 14 robot runs. The mean of a series is depicted using a black point, while the median is drawn as a line through the center of the box. The box represents the middle 50 percent of the data. At both sides it is connected with the last data point within the 1.5* interquartile range from the first resp. third quartile. Data points outside are defined as (°) outliers.

**Figure 5:**
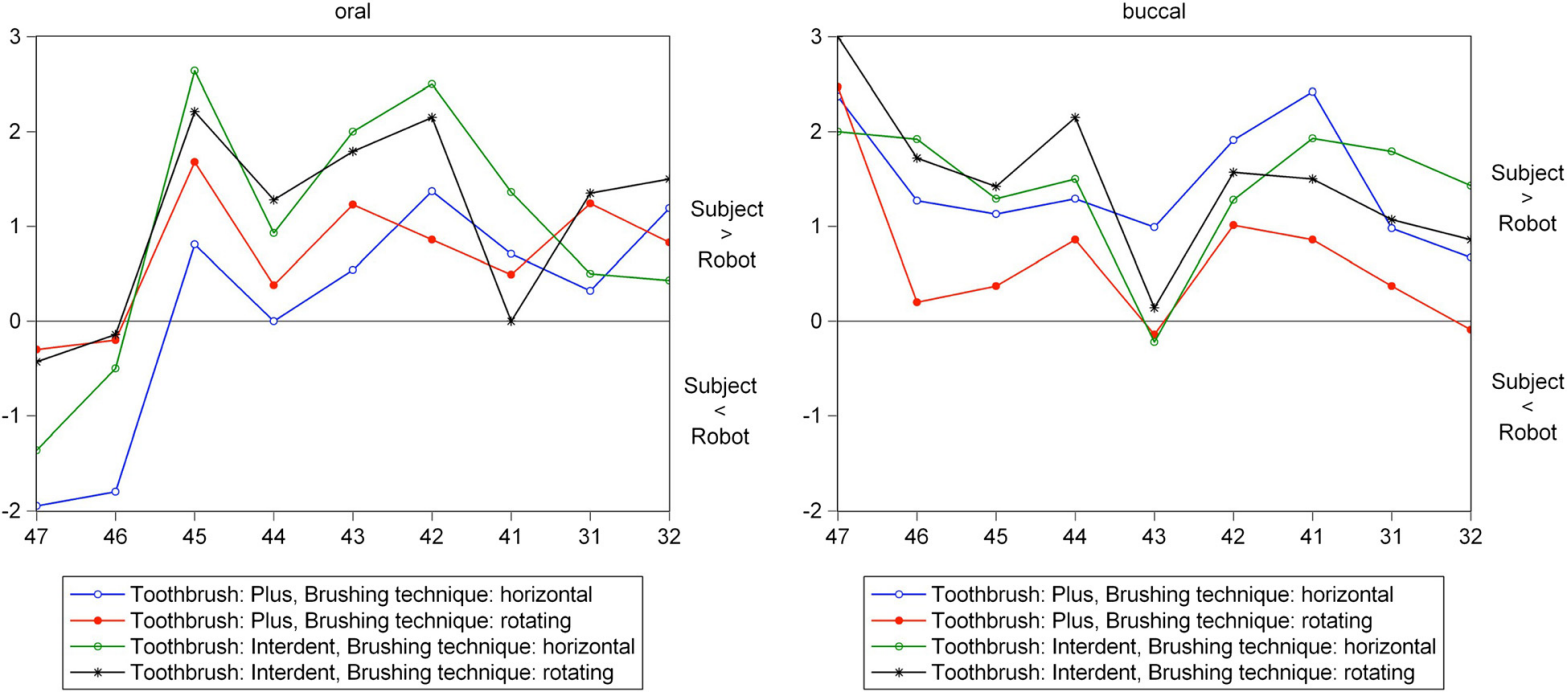
**Correlation of not cleaned planimetrical fields (range of 0–9 fields) in clinical versus robot tests.** Cleaning patterns tooth by tooth 32 to 47, toothbrush A (Plus) versus toothbrush B (Interdent). Robot cleaning efficacy is slightly higher compared to the cleaning efficacy of the subjects (except for tooth 46 and 47 orally), mean values of number of oral (left) and buccal fields (right) with residual plaque. Number of observations: n = 27 clinical study; n = 14 robot study. Spearman rank correlation coefficients: Oral: B(hor) vs. B(rot): r = 0.62 (0.05 < p < 0.10). B(hor) vs. A(hor): r = 0.68 (0.01 < p < 0.05). B(hor) vs. A(rot): r = 0.80 (p < 0.01). B(rot) vs. A(hor): r = 0.73 (0.01 < p < 0.05). B(rot) vs. A(rot): r = 0.87 (p < 0.01). A(hor) vs. A(rot): r = 0.83 (p < 0.01). Buccal: B(hor) vs. B(rot): r = 0.80 (p < 0.01). B(hor) vs. A(hor): r = 0.48 (p > 0.10). B(hor) vs. A(rot): r = 0.73 (0.01 < p < 0.05). B(rot) vs. A(hor): r = 0.45 (p > 0.10). B(rot) vs. A(rot): r = 0.77 (p < 0.01). A(hor) vs. A(rot): r = 0.60 (0.05 < p < 0.10).

The total plaque removal efficacy of robot toothbrushing was significantly higher compared to clinical toothbrushing using the same test brushes, the same brushing movements and force and assessing the same teeth. However, both molars exhibited at the oral sites more residual plaque compared to the clinical data (Table 1). Nevertheless, the significant index differences robot vs. subjects were rather small given the planimetrical plaque index values per tooth site of max 9.00 (all fields exhibit residual plaque) and min 0.00 (no one field does exhibit residual plaque). Clinical and robot data followed the same direction of tooth specific cleaning efficacy as well as the site-specific differences between lingual (oral) and buccal smooth surfaces and planimetrical risk fields at the gum line (planimetrical fields A, B and C) and interproximally in-between the teeth (planimetrical fields D and F). Figure 4 illustrates the parallel plaque removal efficacy documented for the assessment of subjects, and of robot teeth. The following Figure 5 represents the tooth-specific parallel differences of cleaning efficacy robot vs. subjects. The Spearman rank correlation coefficient approved the high correlation of differences in the tooth by tooth cleaning outcome of subjects and of the robot (Figure 5). Finally, the multivariate analysis revealed no statistically significant differences of plaque removal efficacy by different brushing movements in both clinical and robot programmes.

OLS estimation with remaining plaque (PLAQUE) as an endogenous variable for clinical and robot data has the following form (see [18] and [19] for multivariate analysis):

$$\begin{array}{ll} \mathrm{PLAQUE} & =C+\pi_{1}\mathrm{BMH}+\pi_{2}\mathrm{TBA}+\pi_{3}\mathrm{LING}\\ & +\pi_{4}\mathrm{ABS}1+\pi_{5}\mathrm{ABS}2+\epsilon \end{array}$$

with horizontal brushing movement (BMH), the use of toothbrush A (TBA), the lingual site (LING), incisors and canines (ABS1) and premolars (ABS2) as dummy-regressors.

Table 2 shows the estimated coefficients and significant test values. The results of OLS-estimation of residual plaque in clinical and robot study agree in the main results: The type of brushing movement is insignificant for the extent of residual plaque. Toothbrush A significantly leads to more residual plaque than toothbrush B. Oral sites were significantly better cleaned than buccal sites and residual plaque is significantly higher at molars.

**Table 2:** Multivariate OLS-analysis of residual plaque in clinical and robot study – Estimated Coefficients

	**Plaque (Clinical data)**	**Plaque (Robot data)**
**C (Constant)**	**36,13** (1945***)	**29,03** (30,92***)
${\overset{-}{\Pi }}_{1}$ **(BMH)**	**0,01** (0,00)	**−1,59** (−2,07*)
${\overset{-}{\Pi }}_{2}$ **(TBA)**	**6,74** (4 45***)	**5,87** (7,66***)
${\overset{-}{\Pi }}_{3}$ **(LING)**	**3,95** (2,61**)	**8,59** (11,20***)
${\overset{-}{\Pi }}_{4}$ **(ABS1)**	**−15,57** (−8,38*** )	**−12,92** (−13,76***)
${\overset{-}{\Pi }}_{5}$ **(ABS2)**	**−9,92** (−5,34***)	**−8,47** (−9,02***)
**mean**	32,98	28,33
**sd**	8,36	7,85
**R**^**2**^	0,85	0,96
**DW**	1,88	2,16
**BPG**	3,33	2,90

**Explanations:** Number of observations: n = 24. π_**1, …,**_ π_**5**_: Estimated coefficients of exogenous variables.

BMH: Horizontal brushing movement, TBA: Toothbrush A, LING: Lingual site, ABS1: Incisors and canines, ABS2: premolars. t-values in parentheses. (*), (**), (***): the null hypothesis “estimated coefficient is not different from zero” can be rejected at a significance level of 10, 5 resp. 1 percent. R ^2^: coefficient of determination. DW: Durbin-Watson test for autocorrelation of the residuals. BPG: Breusch-Pagan-Godfrey test for heteroscedasticity.

In particular, the estimated coefficients for the toothbrush are of great importance. In both cases (clinical and robot data), they significantly differ from zero with same sign. In the estimation of the clinical data, the use of toothbrush A leads to 6.74% more plaque than the use of toothbrush B (t = 4.45 (p < 0.01)). In the robot study it is 5.87%, also highly significant (t = 7.66 (p < 0.01)). The clinical results in favour of toothbrush B are reproduced in the robot study.

## Discussion

Laboratory testing of cleaning efficacy of different toothbrush designs is essential for the development of new prototypes as well as for the consumers interest in improving their individual oral hygiene. Therefore, any laboratory testing should be as close as possible to the real clinical conditions. In this sense, the robot testing approach has many advantages. This includes the programming (“teaching the robot”) and standardization of any brushing movement, the calibration of different brushing forces and brushing time. Planimetrical plaque index systems are applicable, and exploratory tests with 5 runs or statistical valid tests with 7 runs per test brush, per test movement etc. are possible. However, the clinical validation of the rather complex robot testing programme is a prerequisite for any clinical relevant conclusion concerning the plaque-biofilm controlling efficacy of toothbrushes. It was, therefore, the primary aim of the study to develop and validate a robot toothbrushing test in two steps.

The first step was the standardization of most recommended toothbrush movements of a common brushing force and brushing time and of a sensitive planimetrical plaque assessment index system applicable in the clinical and robot setting. Then, the translation of all this clinical parameters to the robot programme followed.

The second step was the comparison of clinical and robot plaque removal efficacy and the statistical approval whether the two data sets do correlate to declare the robot clinically validated.

The number of subjects was within the requirements for clinical toothbrush tests according to the ADA Acceptance Program Guidelines [20]. The used brushing force of 3.5 N was slightly higher than the mean brushing force of uninstructed adults of 2.3 N (+/− 0.7) but within the range of an acceptable force of 4.0 N [21,22]. The brushing time of 20 s buccally and 20 s orally per two sextants would sum-up in a total brushing time of 120 s for the complete dentition. This is slightly above a mean brushing time of uninstructed subjects of 96 s +/− 36 s [22].

All in vitro tests developed so far are capable to measure special aspects of plaque removal like inter-proximal penetration, brushing forces, brushing time and brushing technique, but lack the simulation of oral biofilms on tooth surfaces [2,3,7,10]. None of the robot testing methodologies developed so far could satisfactorily reproduce the complex human tooth cleaning behaviour of rather sticky plaque biofilms.

According to the most comprehensive study of Claydon et al. of comparative professional plaque removal (using 8 branded toothbrushes) there was a clear tooth-specific and site-specific removal efficacy, but no significant differences between upper and lower arches [23]. Consequently, in the present study two sextants of lower teeth including incisors, canines, premolars and molars were postulated to be representative for the whole dentition. The left lower canine and the right lower wisdom tooth were present but not scored to maintain the interproximal planimetrical fields. Claydon et al. did also show that there were no significant differences between the 8 toothbrushes. It supports also “the conclusion that the user is by far the most significant variable” [23].

The tooth-specificity and site-specificity of plaque accumulation and removal is caused by the size and morphology of teeth and by anterior vs. posterior teeth. Individual patterns may also be influenced by eugnathic vs. dysgnathic dentitions. The mean values of plaque removal tooth by tooth and site by site in the clinical study arm corresponded significantly with the mean values of the robot arm. However, the cleaning efficacy is significantly higher after robot brushing compared to clinical brushing. Therefore, the robot programme avoids any individual adaptation of brushing movements and brushing force different from the set standard. To test the brushing efficacy as such, the robot and the standardized programme seems to be superior to clinical testing as long as the toothbrush head design, the number and size of filaments, their stiffness and direction is concerned.

Abrasion, attrition and erosion, the tooth wear, is physically different from gentle brushing actions. So, a toothbrush is aimed at brushing (not abrading, not eroding) tooth biofilms partly away to control plaque accumulation. Rosema et al. have recently documented that “dentifrice did not show an added effect on instant plaque removal efficacy” using new and used manual toothbrushes [24]. These results and other clinical studies [23] support the approach in the present study not to use dentifrice while searching for plaque or simulated plaque removal. The robot toothbrushing technology can be employed with gingival masks simulating the permanent and mixed dentitions and, by adapting the planimetrical index, without masks simulating gingival recession [25]. Recently, the robot testing of children’s toothbrushes in the deciduous dentition has been clinically validated [26].

Due to short life cycles of new toothbrush models there is a need for quick and reliable testing of different prototypes and new models. One requirement before manufacturing a new toothbrush model is, that it should be at least as effective in plaque removal as its predecessor. Clinical tests normally cannot discriminate small differences between toothbrush models and therefore are not ideal in the early stage of development. The main advantage of the proposed robot test is the reproducibility of the test conditions and the precise data recording. Because of the clinical validation the robot methodology can predict clinical toothbrushing efficacy.

The individual data sets concerning the risk of residual plaque at teeth at risk, sites at risk and planimetrical fields at risk can be accumulated in databanks where they are comparable because of the standardization for many different forms, toothbrush prototypes and models on the market.

## Conclusion

Planimetrical plaque scoring at all teeth of a representative artificial permanent dentition has been clinically validated. Therefore, the new robot test of toothbrushing can be recommended for the reproducible evaluation of plaque control and cleaning efficacy of different toothbrush designs and brushing actions.

## Competing interests

The authors declare that they have no conflict of interests. This study was supported by GlaxoSmithKline and M + C-Schiffer.

## Authors' contributions

TL did planing the project, transferred clinical data to the robot programme and contributed to writing the manuscript; SS was responsible for the clinical programme; BJ did the statistics; PG was supervising the project and wrote the manuscript. All authors read and approved the final manuscript.

## Pre-publication history

The pre-publication history for this paper can be accessed here:

http://www.biomedcentral.com/1472-6831/14/82/prepub
